# Oxytree Pruned Biomass Torrefaction: Process Kinetics

**DOI:** 10.3390/ma12203334

**Published:** 2019-10-12

**Authors:** Kacper Świechowski, Sylwia Stegenta-Dąbrowska, Marek Liszewski, Przemysław Bąbelewski, Jacek A. Koziel, Andrzej Białowiec

**Affiliations:** 1 Faculty of Life Sciences and Technology, Institute of Agricultural Engineering, Wrocław University of Environmental and Life Sciences, 37/41 Chełmońskiego Str., 51-630 Wrocław, Polandandrzej.bialowiec@upwr.edu.pl (A.B.); 2 Faculty of Life Sciences and Technology, Institute of Agroecology and Plant Production, Wrocław University of Environmental and Life Sciences, 24A Grunwaldzki Sqr., 53-363 Wrocław, Poland; marek.liszewski@upwr.edu.pl; 3 Faculty of Life Sciences and Technology, Department of Horticulture, Wrocław University of Environmental and Life Sciences, 24A Grunwaldzki Sq., 53-363 Wrocław, Poland; przemyslaw.babelewski@upwr.edu.pl; 4 Department of Agricultural and Biosystems Engineering, Iowa State University, Ames, IA 50011, USA; koziel@iastate.edu

**Keywords:** Oxytree, Paulownia, torrefied biomass, pruned biomass, valorization, renewable energy, fast-growing biomass, energy crops, brownfields, kinetics parameters, activation energy

## Abstract

Oxytree is a fast-growing energy crop with C4 photosynthesis. In this research, for the first time, the torrefaction kinetic parameters of pruned Oxytree biomass (*Paulownia clon* in Vitro 112) were determined. The influence of the Oxytree cultivation method and soil class on the kinetic parameters of the torrefaction was also investigated. Oxytree pruned biomass from a first-year plantation was subjected to torrefaction within temperature range from 200 to 300 °C and under anaerobic conditions in the laboratory-scale batch reactor. The mass loss was measured continuously during the process. The relative mass loss increased from 1.22% to 19.56% with the increase of the process temperature. The first-order constant rate reaction (*k*) values increased from 1.26 × 10^−5^ s^−1^ to 7.69 × 10^−5^ s^−1^ with the increase in temperature. The average activation energy for the pruned biomass of Oxytree torrefaction was 36.5 kJ∙mol^−1^. Statistical analysis showed no significant (*p* < 0.05) effect of the Oxytree cultivation method and soil class on the *k* value. The results of this research could be useful for the valorization of energy crops such as Oxytree and optimization of waste-to-carbon and waste-to-energy processes.

## 1. Introduction

It is estimated that the amount of bio-renewable energy in the European Union (EU) will continue to grow by ~22 EJ∙year^−1^ from the year 2010 to 2030. This increase is due to the 2009/28/EC directive adopted by the EU [1]. It has been shown that, in 2020, biomass will account for almost 60% of the total renewable energy produced in the EU [2]. The last update of the EU targets and the share of renewable energy sources in the total energy consumption was 27%, contributing to the increasing demand for wooden biomass [3]. The forecasted growth of the biomass share in the energy production balance indicates the importance of the research on new, fast-growing energy crops, especially those which can be cultivated in weak or degraded soils.

To date, short rotation plantations of wood, such as willow, poplar, black locust, alder [4], and aspen [5], were investigated. All mentioned plant species are C3 photosynthesis plants [6,7]. It is known that a more efficient pathway is photosynthesis C4. It is assumed that, for some plants, successful conversion of the C3 photosynthesis pathway to C4 could result in an increase in crop yield by 50–60% [8,9]. *Paulownia* sp. genus developed the C4 photosynthesis [10]. An additional advantage of *Paulownia* trees is the ability to adapt to different climatic conditions, high mass yield [11,12], and the possibility of establishing plantations on wasteland, brownfields, as well as degraded lands [13].

One of the clones of *Paulownia* is *Paulownia clon* in Vitro 112, which can be characterized by increased biomass, reaching a height of 16 m at the age of 6 y. The Oxytree wood is utilized in construction or furniture-making [14]. *Paulownia clon* in Vitro 112 ranks as class I on Janka’s hardness scale—very soft wood [15]. It has been shown that the biochar from *Paulownia* produced at 600 °C is a pure carbon without organic composition, characterized by a pore size from 35.8 to 290.5 μm. Such material, after proper treatment, can be applied as a filtration agent or catalyst [15]. Oxytree can also be utilized for energy purposes. The high heating value in dry mass is 19 470 J∙g^−1^, with low ash (1.29%) and chlorine (0.01%) content [16].

Bulky biochar can be pelletized for energy content densification. However, the main limitation of the classic pelletizing is the necessity of biomass drying as well as the high energy demand on its grinding [17]. The problem of moisture and grinding of biomass can be solved by using low-temperature pyrolysis, also known as torrefaction. The resulting solid material is characterized by higher energy content and improved fuel properties, which have been widely described in the case of other types of wooden biomass [18,19]. The additional benefit of torrefaction is the decrease of the energy demand for grinding from approximately 240 kW∙Mg^−1^ to 20–80 kW∙Mg^−1^ [20]. Torrefaction is a thermochemical pretreatment process that improves biomass utilization values. During torrefaction, the biomass is treated with temperatures of 200–300 °C in an inert condition for up to 1 h [21].

Kinetics are important for biochar valorization. Since biomass is a complex mixture of biopolymers, during the pyrolysis/torrefaction, the thermal decomposition of biomass takes place in multiple steps, which can overlap with each other. Kinetics studies are essential for the understanding of occurring reactions. The term ‘kinetics’ pertains to kinetics parameters such as the activation energy, pre-exponential factor, reaction rate, and others. The kinetics are used for the design and optimization of reactors, and to establish optimal process conditions. The kinetics of solid torrefied biomass can be obtained in many ways, based on analyses, such as thermogravimetric analysis (TGA), differential thermal analysis (DTA), or differential scanning calorimetry (DSC). The determination of kinetics has two stages. First is an experiment involving one of the analyses mentioned above, and the second stage is based on mathematical analyses of experimental data [22].

Recently, we [23] presented results of the influence of the pruned Oxytree biomass torrefaction temperature and duration on the proximate and ultimate properties of the produced biochar. However, to date, no scientific papers have presented systematic research on the kinetics of torrefaction of *Paulownia*, including the pruned biomass of Oxytree. The kinetics and activation energy of pruned biomass of Oxytree torrefaction is unknown but is essential for modeling the process efficiency and energy demand.

**Figure 1:**
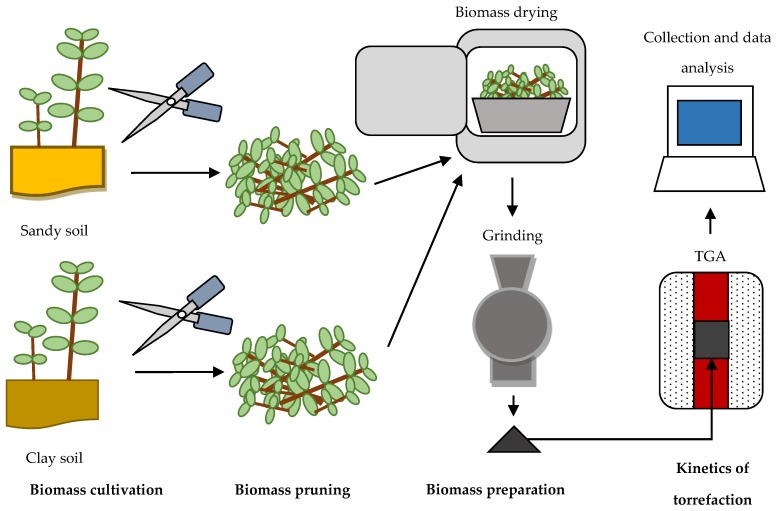
Scheme of experiments to determine the process kinetics of torrefaction for Oxytree prunings.

The aim of the study was the determination (for the first time) of the kinetics parameters and the activation energy of torrefaction of pruned biomass of Oxytree corresponding to the agronomic cultivation conditions and soil type. The scheme of the experiment is shown in Figure 1. The TGA analysis in isothermal conditions and first-order reaction models were used to determine the activation energy.

We hypothesized that the soil type and Oxytree cultivation method could impact the torrefaction kinetics as it is generally known that the composition of biomass can be affected by soil or cultivation methods. Thus, this research focuses on pruned Oxytree biomass produced in different soil types and under different cultivation methods (irrigation/no irrigation, and geotextile/no geotextile). Rodrigues et al. [24] showed that soil composition correlated with biomass fuel quality. This means the soil influences biomass chemical composition. Similarly, Achinelli et al. [25] showed that irrigation has a statistically significant impact on the higher heating value (*HHV*) of biomass (willows). On the other hand, irrigation did not have an impact on ash content, vessel diameter, fiber wall area, and fiber wall thickness [25].

## 2. Materials and Methods

### 2.1. Oxytree Biomass Samples

In a previous data descriptor article [26] titled “Fuel Properties of Torrefied Biomass from Pruning of Oxytree” raw data with descriptions about biomass acquisition, biomass properties, production technology (soil type, mineral fertilization, irrigation), the process of torrefaction, and properties of torrefied biomass were presented in detail. In brief, Oxytree pruned biomass came from two experimental plantations in Poland. The biomasses differed by agro-technical cultivation practices (geotextile and irrigation) and soil type. The cultivation scheme is presented with the following symbols, as is presented below [26]:

- S(G−)(I−),
- S(G+)(I−),
- S(G−)(I+),
- S(G+)(I+),
- C(G+)(I−),
- C(G−)(I−),
- C(G+)(I+),
- C(G−)(I+),

For these symbols, S and C letters stand for the soil type, i.e., S—sandy soil, classified as V soil belonging to brunic arenosols, C—clay soil classified as Phaeozems, respectively, based on the FAO World reference database for soil resources (2014) [27]. The sign (+) stands for cases where geotextile/irrigation, G/I, was used, and (−) for when geotextile/irrigation was not used during cultivation [26].

Oxytree shoots propagated to ~20–40 cm were planted with 4 m × 4 m (16 m^2^ per tree) spacing on 19 May 2016. The trees subjected to analysis were pruned on 27 September 2016. The analyzed trees represent pruned biomass after one year of vegetation. A typical agro-technical care treatment takes place 12 months after planting in May, i.e., in the next calendar year. This agro-technical care treatment involves trimming 0.05 m above the pitch to derive one main shoot, which then becomes the main trunk of the tree. The examined biomass samples were about 71% of leaves and 29% of shoots of the total fresh mass, respectively (or 66% and 34%, respectively, on a dry mass basis) [26].

### 2.2. Ultimate and Proximate Analysis of Samples

The proximate analyses were conducted using standard methods that included:

- Moisture content determined in accordance with [28], using a laboratory dryer (WAMED, model KBC-65W, Warsaw, Poland),
- Organic matter determined in accordance with [29], using a muffle furnace (SNOL, 8.1/1100, Utena, Lithuania).
- Combustibles and ash content determined in accordance with [30], using a muffle furnace (SNOL, 8.1/1100, Utena, Lithuania),
- Higher heating value (*HHV*) and low heating value (*LHV*) determined in accordance with [31] using calorimeters (IKA POL, model C 200, Warsaw, Poland).

The ultimate analyses were the determination of elemental composition (C, H, N, S, O). Carbon, H, and N contents were determined with an elemental CHNS analyzer (CE Instruments Ltd., Manchester, UK). Sulfur was determined by the atomic emission spectrometry method with excitation in inductively-coupled plasma (ICP-AES) after microwave mineralization, using an atomic emission spectrometer (iCAP 7400 ICP-OES, Thermo Fisher Scientific, Waltham, MA, USA).

### 2.3. Thermogravimetric Analysis—Experimental Design and Procedure

The thermogravimetric analysis (TGA) was carried out using a stand-mounted tubular furnace (Czylok, RST 40x200/100, Jastrzębie-Zdrój, Poland) previously described by Stępień et al. [32].

The mass losses during the torrefaction process were tested under isothermal conditions at 200 °C, 220 °C, 240 °C, 260 °C, 280 °C, and 300 °C with a residence time of 60 min and a CO_2_ flow rate of 10 dm^3^∙h^−1^ to ensure anaerobic conditions. Three repetitions for each temperature and cultivation type were completed. The materials before the tests were dried in a laboratory dryer (WAMED, model KBC-65W, Warsaw, Poland) for 24 h at 105 °C. Before the experiment was started, the empty tubular furnace was heated to the desired temperature. The samples, with a weight of 3 g, were placed in the steel crucible, which was placed in the heated tubular furnace for 1 h. The measurement of mass loss for each temperature was made using a balance coupled to a cuvette with a biomass sample. The measurement of mass loss took place with a 10 s interval and 0.01 g accuracy.

### 2.4. Data Analysis

The obtained TGA data were used to determine the constant reaction rate (*k*, or *k* value) for particular torrefaction temperatures and Oxytree cultivation types. The *k* values were estimated according to [33] to the first-order reaction, Equation (1):

(1) $$m_{s}=m_{o}\cdot e^{-k\cdot t}$$

where:

*m_s_*—mass after torrefaction time *t*, g;
*m_o_*—initial mass, g;
*k*—a constant rate of the reaction, s^−1^;
*t*—time, s.

The estimation of *k* values was completed by nonlinear regression and the Statistica 13.3 software (StatSoft, Inc., TIBCO Software Inc., Palo Alto, CA, USA). Then, Arrhenius plots [34] were created, i.e., ln(*k*)[*T*] vs. 1/*T*, where *k* is a constant rate of the reaction, s^−1^, *T* is the torrefaction temperature, K. A linear trend line was then obtained for Arrhenius plots:

(2) y=a·x+b

Then, the activation energy (*E_a_*) values [33] were determined using coefficient ‘a’ from Equation (2):

(3) $$E_{a}=a\cdot R$$

where:

E_a_—activation energy, J∙mol^−1^;
*a*—the slope coefficient of the linear Equation (2), *k*;
*R*—gas constant, J∙mol^−1^∙K^−1^.

### 2.5. Statistical Analysis

The study of the normality of the relative mass loss (Δ*m*) and *k* values distribution was performed graphically using a quantile chart (Q-Q) and Shapiro–Wilk (S.W.) and Kolmogorov–Smirnov (K.S.) tests along with the Lilliefors correction for a confidence level of α = 0.05. Since the Δ*m* and *k* values were not normally distributed, the analysis of variance was carried out with the non-parametric Kruskal–Wallis (K.W.) test at the α = 0.05 level of confidence for the following groups:

- Δ*m* and *k*—the variable grouping by the cultivation type;
- Δ*m* and *k*—the variable grouping by torrefaction temperatures;
- Δ*m*—the variable grouping by the torrefaction time.

The post-hoc tests were performed for each K.W. test. A lack of statistically significant differences was marked with the same letters on a box plot. All results of statistical evaluation and interpretation are presented in Appendix A (Figure A1, Figure A2, Figure A3, Figure A4, Figure A5, Figure A6, Figure A7, Figure A8 and Figure A9).

## 3. Results

### 3.1. Oxytree Biomass Characterization

The organic matter content for Oxytree was 89.22–91.46% d.m. The flammable fraction accounted for 91.22–93.05% d.m. The *HHV* was >17,900 J∙g^−1^, with the highest value for S(G+)(I−) being 18,577 J∙g^−1^ (Table 1).

The Oxytree biomass was characterized by an average C (43.76%), H (6.65%), N (2.3%), S (0.2%), and O (37.32%) content, respectively (Table 2). The highest C content was measured in C(G−)(I+) 45.7%, and the lowest S content in S(G−)(I−) 0.17%.

### 3.2. Relative Mass Loss During Torrefaction of Oxytree

A loss of mass occurred during torrefaction for all examined Oxytree samples (Table 3). An increase in mass loss corresponded to the increase of the torrefaction temperature. The smallest Δ*m* values of 0.1% were recorded at 200 °C (lowest temperature) and 10 min (shortest time) for S(G−)(I−), C(G+)(I−), C(G−)(I−) and C(G−)(I+) samples. The data show the increasing Δ*m* value with temperature and process time to 19.6% (S(G+)(I+), 300 °C and 60 min). Post−hoc tests showed that there was no impact of cultivation type on mass loss (*p* < 0.05) (Figure A7). However, the torrefaction temperature had a significant effect on each *T* tested (*p* < 0.05) (Figure A8). The increasing torrefaction time led (initially) to a significant increase (*p* < 0.05) of relative mass loss (Figure A9). In addition, the detailed raw results of TGA tests may be found in [26]. It has been shown that increasing the torrefaction time and temperature increases weight loss. The use of irrigation allowed for a 1.5% lower weight loss in sandy soil, compared to *Paulownia* grown in clay soil. The highest weight loss was always obtained in the variant S(G+)(I+) regardless of the torrefaction temperature.

### 3.3. Oxytree Torrefaction Kinetics

The *k* values significantly (*p* < 0.05) increased with the torrefaction temperature. The lowest *k* value (*k* = 1.25 × 10^−5^ s^−1^) was observed for 200 °C and in the C(G+)(I−) material, and the highest (*k* = 1.82 × 10^−5^ s^−1^) for S(G+)(I+) (Table 4). In general, the lowest *k* was observed for the C(G+)(I+) material, i.e., 1.77 × 10^−5^ s^−1^, 2.72 × 10^−5^ s^−1^, and 3.46 × 10^−5^ s^−1^, at 220 °C, 240 °C, and 260 °C, respectively. The lowest *k* value at 260 °C was associated with the S(G−)(I−) material, regardless of torrefaction time. Also, the S(G−)(I−) material in the temperature range of 260–300 °C had the lowest *k* values of 3.24 × 10^−5^ s^−1^, 4.69 × 10^−5^ s^−1^, and 6.42 × 10^−5^ s^−1^, respectively.

The highest values of the constant rate *k* for the 220–260 °C range were recorded for the C(G−)(I−) material and were 2.3 × 10^−5^ s^−1^, 3.44 × 10^−5^ s^−1^, and 4.63 × 10^−5^ s^−1^ respectively. The highest *k* value at 280 °C was observed for S(G−)(I+), and at 300 °C for S(G+)(I+). Post-hoc tests showed that there were no statistically significant differences (*p* < 0.05) between the impact of a particular cultivation type and resulting *k* value. The statistical significance (*p* < 0.05) is marked with letters in Figure A4.

The S(G+)(I+) material had the highest observed activation energy value of *E_a_* = 39,282 J·mol^−1^, while the smallest (*Ea* = 33,369 J·mol^−1^) was associated with the S(G+)(I−) material. The average value of *E_a_* for all type of pruned Oxytree was 36,510 J·mol^−1^. The coefficient of determination R^2^ for each *E_a_* value was >0.98, which indicates a high matching degree of the estimated parameters to the experimental data. Since the cultivation type did not have an impact on the *k* value, it can be assumed that cultivation type did not have an impact on the energy activation, and there are no statistically significant differences in the determined energy activation (*p* < 0.05).

The Arrhenius plots for each cultivation type are presented in Appendix B. Figure A15, Figure A16, Figure A17, Figure A18, Figure A19, Figure A20, Figure A21 and Figure A22 illustrate linear models for the activation energy estimation. The plots of mass loss at setpoint temperatures are presented in Figure A23, Figure A24, Figure A25, Figure A26, Figure A27 and Figure A28 as the comparison between experimental and model kinetics. No apparent differences (>5%) were observed between the experimental and calculated mass loss.

## 4. Discussion

### 4.1. Oxytree Biomass Sample Characterization

Woody biomass is composed of three basic elements (C, 52%; H, 6%; O, 42%) [35]. This means that the tested Oxytree biomass has a lower content of C and O by ~8% and ~5%, respectively. The C and H content in the fuel positively influences the energy value of biomass. Oxygen contained in biomass favors burning processes. On the other hand, it reduces its energy value [36].

Pruned biomass of Oxytree was characterized by the organic matter content of ~90%, combustibles content of ~92%, ash ~8%, and the *HHV* 18.3 MJ∙kg^−1^ (Table 1). As for woody biomass, the material tested had a high ash content and it is desired for energy crops to be low. For example, in the case of willow and poplar, ash accounts for 0.5–1.2% [37]. Previous research showed that *Paulownia tomentosa* has a 0.19–6% ash content depending on the plant part, with 6% in the leaves and 0.23% in the wood [38]. High ash content in the tested Oxytree material can be caused by a high leaf content of 66% compared to 34% being shoots.

The examined pruned biomass of Oxytree had about 4% more combustibles, compared to the *Paulownia tomentosa* wood from a research forest of Kangwon National University, Republic of Korea [38]. The mean *HHV* was 18,295 ± 31; slightly below values in the literature. The *HHV* depends on the *Paulownia* species (grown in Croatia) with an average of ~19 MJ∙kg^−1^ [39]. For comparison, the *HHV* of willow and poplar (at a plantation located in Northeastern Poland) is about 19.5 MJ∙kg^−1^ [40] and 18.5–19.1 MJ∙kg^−1^, respectively [41]; confirming that Oxytree energy concent is comparable to conventional woody biomass considered for energy crops.

The determined mean concentrations of elements of the studied Oxytree were similar to those reported for other *Paulownia* species. *Paulownia elongata* (at a plantation located in the West Black Sea region of Turkey) had 45.8% C, 6.3% H, and 0.4% N content [42]. The tested biomass was characterized by a higher N content of 2.3% (Table 2), which can be an effect of N mineral fertilization applied 40 kg·ha^−1^ (pre-planting) and doses of 20 kg·ha^−1^, supplied monthly [26]. Vusić et al. [39] tested three *Paulownia* species which showed similar contents of C, H, N, S, and O at 49% C, 5.8% H, 0.2% N, 0.05% S, and 44% O, respectively. In comparison to these data, the Oxytree tested here had ~5% less C and ~12% O. Noticeable discrepancies in the results observed by other authors may be results of differing conditions of plant growth in different climates.

The application of the soil cultivation method that is appropriate to the soil type significantly influences (*p* < 0.05) the content of organic matter in both sandy and clay soils (Figure A10). The content of organic substances was the lowest in variants without the support of soil cultivation and was lower by ~1% compared to other variants, and very similar in both soil types at ~89.5% d.m. The highest content of organic matter content was noted in clay soil without geotextile (C(G-)(I+)); however, the use of irrigation had a significant difference (*p* < 0.05) compared with other variants (Figure A10).

The lowest ash content was obtained by cultivating *Paulownia* in clay soil (7.87% d.m.) than in sandy soil (8.26% d.m.). However, the highest ash content was obtained for the variant of clay soil without geotextile and with irrigation (C(G-)(I+))—statistically different (*p* < 0.05) from the other variants (Figure A11). Similar results were obtained in the case of combustible content. The best variant was cultivation in clay soil with a geotextile and no irrigation (C(G+)(I−)), i.e., 93.25% combustible content (Figure A12). This result could be explained by the high compactness of such (clayey) soils, where the irrigation procedure improves the oxygenation of the roots, allowing better plant growth [26].

Although the best conditions for the production of biomass are cultivated in clay soil, it was noted that the highest values of *HHV* and *LHV* were obtained in the sandy soil variant with geotextile and no irrigation (S(G+)(I−)). However, no statistically significant (*p* < 0.05) difference was found between the variants (Figure A13 and Figure A14). The obtained biomass properties are much higher than in the case of *Paulownia* biomass cultivated in Turkey, where the content of moisture and organic substances was lower (by ~3.5%), with much lower ash content of 1.05% d.m. [42], at 5–6% moisture and 8–9% d.m. ash obtained in this study [26].

### 4.2. Mass Loss During Torrefaction of Oxytree

Due to the novelty of the examined materials, there are no existing data to directly compare with (i.e., torrefaction of *Paulownia*). Nevertheless, the *Paulownia clon* in Vitro 112 prunings are an example of woody biomass, and they can be compared to other published data. The increase of relative mass loss due to the increase of torrefaction temperature and time is obvious and expected based on the literature [43,44]. For the examined Oxytree material, the visually noticeable mass loss started after ~10 min. Examined materials after torrefaction at 200–220 °C for 60 min had mass losses in the range 1.2–4.1% (Table 3). The mass losses were between 5.9% and 7.4% for temperatures 240–260 °C. Tested materials at 280–300 °C had mass losses of 11.6–19.8%. The average relative mass loss for tested biomass at temperatures of 260, 280, and 300 °C, and a time of 30 min was 7.0%, 10.5%, and 14.5%, respectively. For the same temperatures and time of 60 min, these values were 9.8%, 13.7%, and 18.4%, respectively (Table 3).

For comparison, torrefaction of Douglas fir sawdust for 30 min and temperatures of 250, 275, and 300 °C caused a relative mass loss of approximately 9%, 19%, and 35%, respectively. For the same temperatures and time in the 60 min process, Douglas fir has losses of 11%, 25%, 48% [45]. Becker and Scherer [46] presented mass losses for pine and beech at temperatures of 240, 270, and 300 °C for 25 min. For pine, these values are 3%, 7%, and 14%, and for beech, 3%, 13%, and 20%, respectively. The presented data show that the tested biomass of *Paulownia clon* in Vitro 112 is characterized by a relatively low value of Δ*m* for a time of 60 min. In the 30 min case, the values of Δ*m* are close to the values from the work of Becker and Scherer [46].

In another study, the relative mass losses during the torrefaction of spruce sawdust at temperatures of 230, 250, 270, and 300 °C, were 14%, 23%, 32%, and 53%, respectively [47]. Similarly, high mass losses of 10%, 21%, 32%, and 44% were also noted for beechwood in 30 min torrefaction under temperatures of 240, 260, 280, and 300 °C, respectively [48]. Observed higher Δ*m* by Wang et al. [47] and Gucho et al. [48] probably resulted from the methodology of the conducted research. In [47] and [48], the ‘time’ refers to the time of active heating (i.e., it does not include cooling time). In this study, measurements of mass losses were stopped after 60 min of active heating, and mass loss during cooling was not measured. It is important to mention that the torrefaction process is still ongoing during cooling until the temperature falls below ~200 °C. This is because the temperature inside of torrefied materials is still higher than ambient temperature [49] and, therefore, the process does not end immediately. Bridgeman et al. [50] included the cooling period in the total time of the torrefaction process.

The noticeable loss of mass in the material started after about 10 min. This means that the tested materials needed ~10 min to warm up in their entire volume. This is a long period if the weight of the sample (3 g) is taken into account. For determination of the influence of the sample mass on the heat transport within the biomass, additional tests of the *Paulownia* heat conductance are warranted. It would be necessary for scaling up the Oxytree torrefaction process.

The statistical tests (K.W.) showed that cultivation type had no effect on the Δ*m* (*p* < 0.05) (Figure A7). The temperature had a significant effect on the Δ*m* value (*p* < 0.05); the post-hoc test showed that the differences occur for each temperature interval (Figure A8). Time also had a significant impact (*p* < 0.05) on the Δ*m* value; the post-hoc test showed that significant differences did not occur between 30 and 50 min and between 40 and 60 min (Figure A9).

### 4.3. Oxytree Torrefaction Kinetics

**Figure A3:**
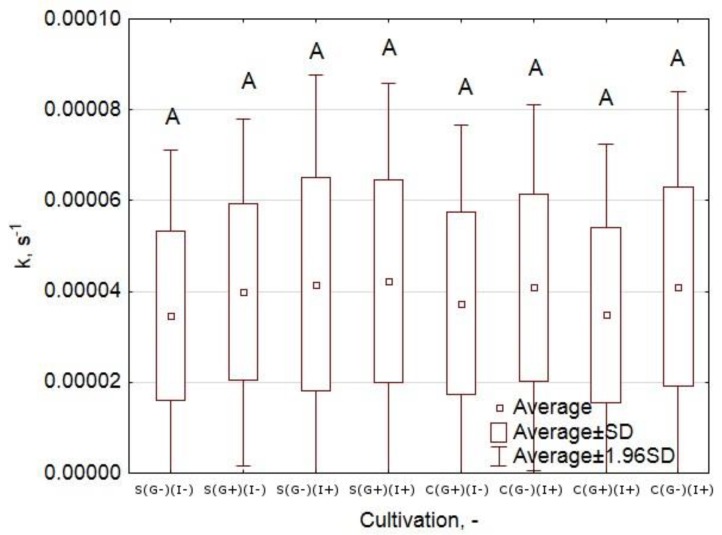
Box plot of *k* in relation to Oxytree cultivation type. Letters present a lack of statistically significant differences (*p* < 0.05) between the studied groups.

**Figure A4:**
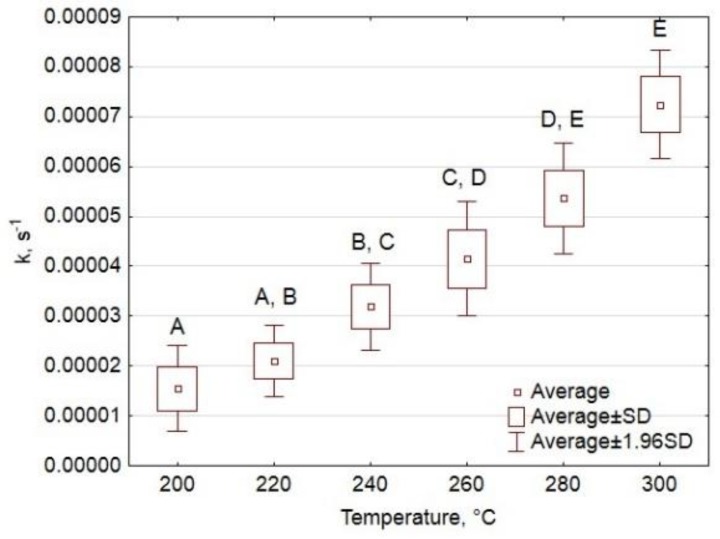
Box plot of *k* in relation to torrefaction temperature. Letters present statistical significance (*p* < 0.05) of differences between the studied groups.

The statistical tests (K.W.) showed that the cultivation method and soil type had no effect on the *k* value (*p* < 0.05) (Figure A3). The temperature had a significant effect on the *k* value (*p* < 0.05); the post-hoc test showed that the differences did not occur for any of the temperature intervals. There were no significant differences between 200 and 220 °C, 220 and 240 °C, 240 and 260 °C, 260 and 280 °C, and 280 and 300 °C. This also means that a statistically significant increase in *k* values occurred every 40 °C (Figure A4).

**Figure A7:**
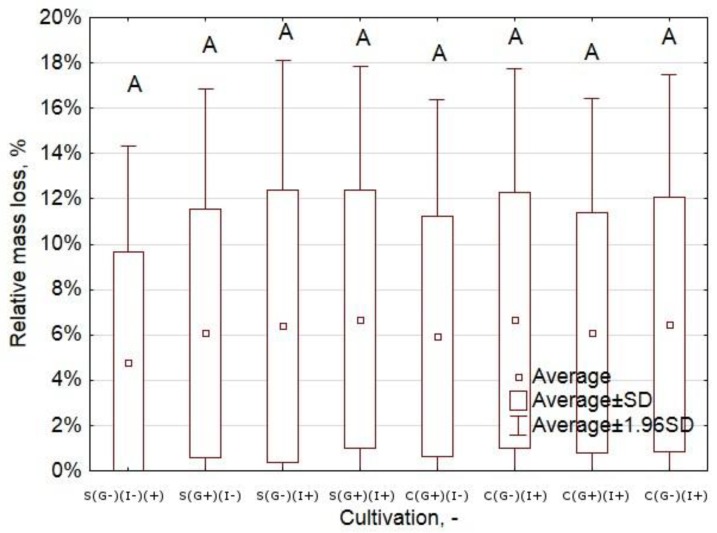
Box plot of Δ*m* during torrefaction in relation to Oxytree cultivation types. Identical letters (A) represent a lack of statistically significant differences (*p* < 0.05) between the studied groups.

**Figure A8:**
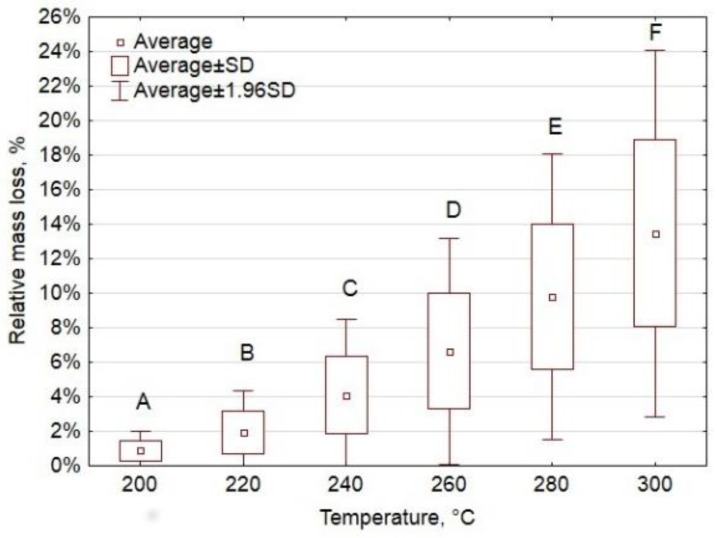
Box plot of Δ*m* during torrefaction in relation to process temperature. Letters present a statistically significance (*p* < 0.05) of differences between the studied groups.

**Figure A9:**
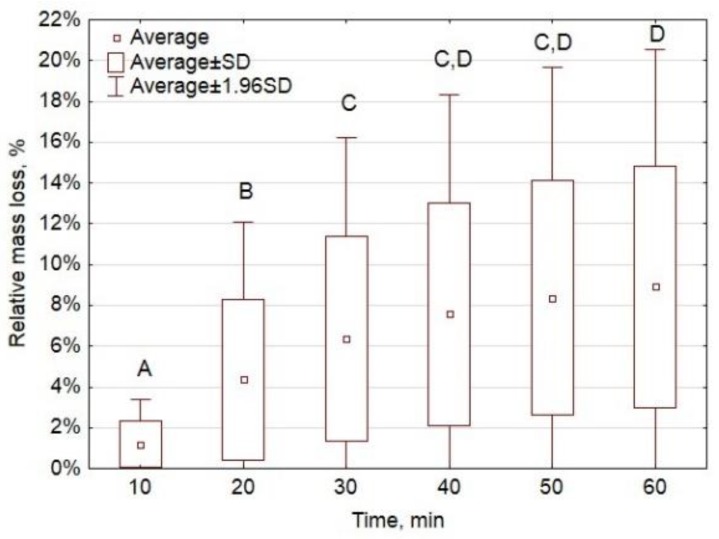
Box plot of Δ*m* during torrefaction in relation to the process time. Letters present statistical significance (*p* < 0.05) of differences between the studied groups.

**Figure A10:**
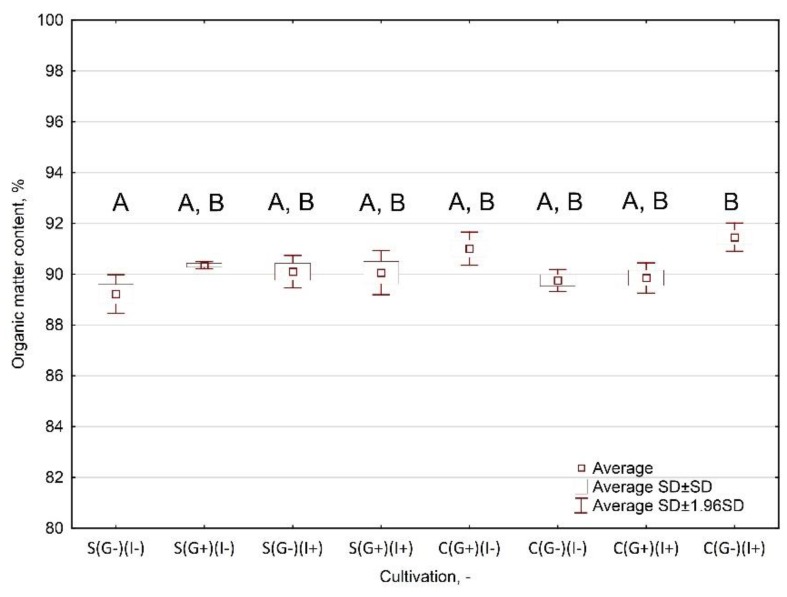
Box plot of organic matter content in raw Oxytree as a function of the cultivation method. Letters present statistical significance (*p* < 0.05) of differences between the studied groups.

**Figure A11:**
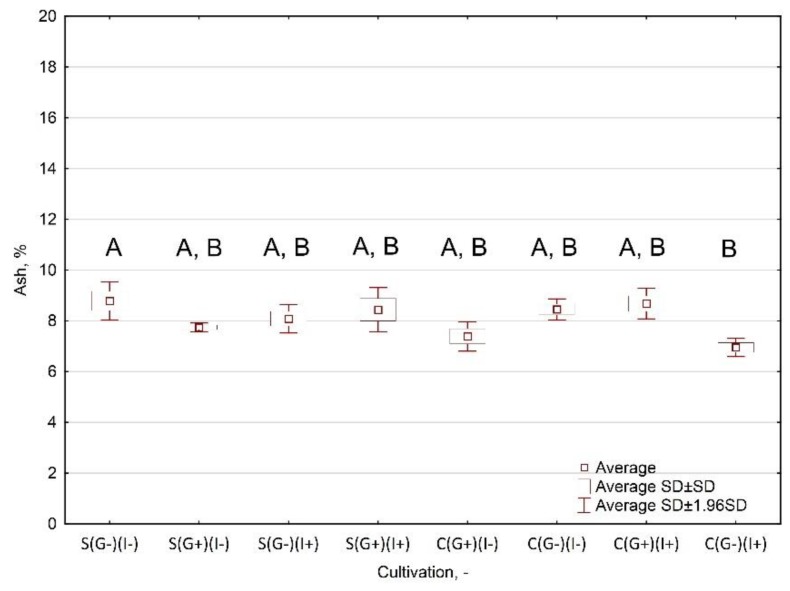
Box plot of ash content in raw Oxytree. Letters present statistical significance (*p* < 0.05) of differences between the studied groups.

**Figure A12:**
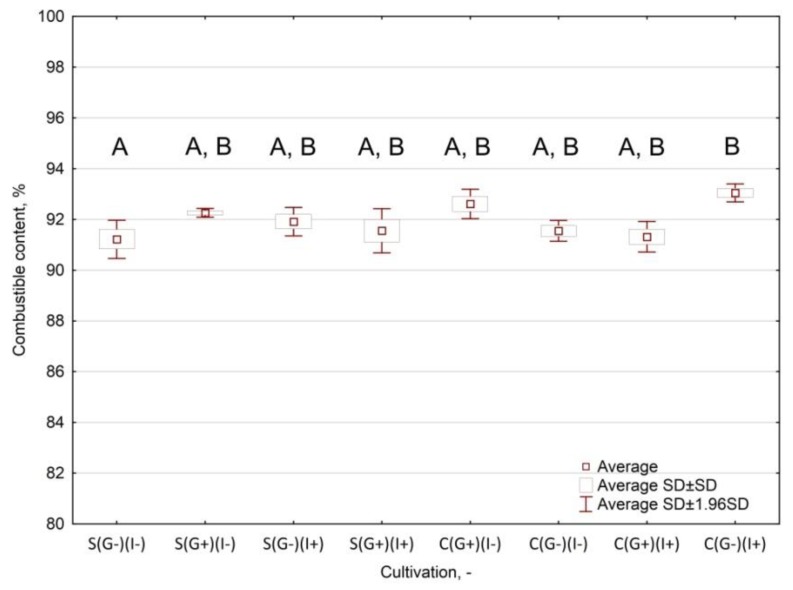
Box plot of combustible content in raw material depending on the cultivation method. Letters present the statistical significance of differences (*p* < 0.05) between the studied groups.

**Figure A13:**
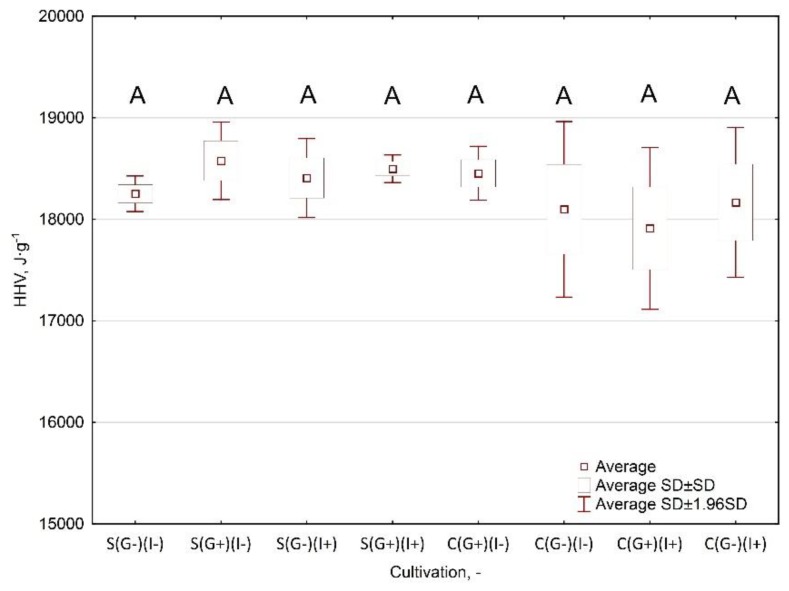
Box plot of *HHV* in raw material depending on the cultivation method. Letters present a lack of statistically significant differences (*p* < 0.05) between the studied groups.

**Figure A14:**
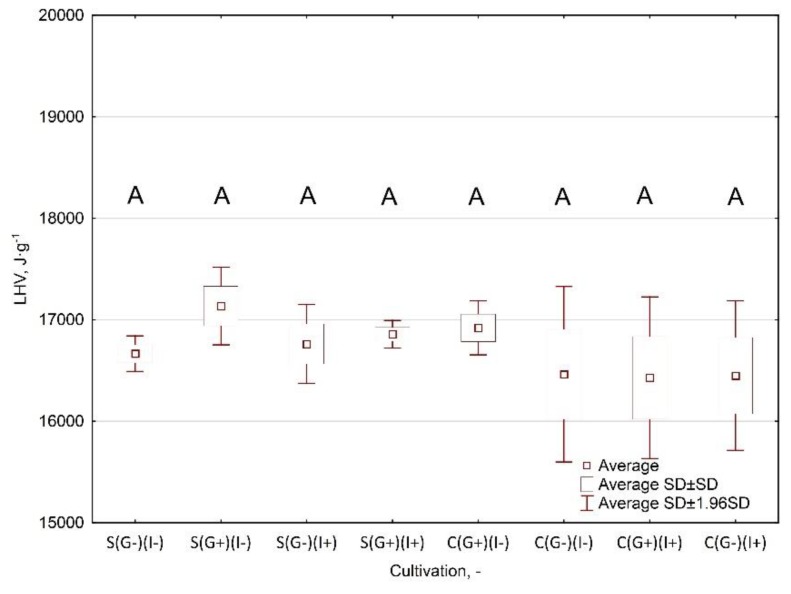
Box plot of *LHV* in raw Oxytree depending on the cultivation method. Letters present a lack of statistically significant differences (*p* < 0.05) between the studied groups.

The K.W. tests indicated that there was no statistically significant (*p* < 0.05) effect of the cultivation method and soil class on the value of relative mass loss (Figure A7) and the constant rate of the reaction *k* for *Paulownia clon* in Vitro 112 (Figure A3). It can, therefore, be assumed that the cultivation method and soil class does not significantly affect the torrefaction activation energy of pruned biomass. This is a very important observation, from a practical point of view, which shows that similar technological parameters of torrefaction could be applied for different Oxytree pruned biomasses. This aspect requires further research in the pilot- and full-scale experiments.

The average *k* values for the examined temperature range of 200–300 °C, with intervals at 20 °C were 1.43 × 10^−5^ s^−1^, 2.04 × 10^−5^ s^−1^, 3.19 × 10^−5^ s^−1^, 4.15 × 10^−5^ s^−1^, 5.37 × 10^−5^ s^−1^, and 7.25 × 10^−5^ s^−1^, respectively. A general trend of the increase in the *k* value was observed along with the increase of the process temperature (Table 4). The same dependence is also reported by Dhanavath et al. [51] for the torrefaction of biomass in general. However, the increase in *k* for Oxytree as a function of temperature was not constant. The largest (*p* < 0.05) differences in *k* values were observed between 280 °C and 300 °C, of 1.88 × 10^−5^ s^−1^, and the smallest between 200 °C and 220 °C, of 6.16 × 10^−6^ s^−1^, but both differences were not statistically significant (*p* < 0.05) (Figure A4).

The value of *k* for Oxytree is challenging to compare with the literature because it depends to a large extent on the kinetic model used to determine it. For example, depending on the test method, the *k* value for 300 °C for oak wood was in the range from 31 × 10^−5^ s^−1^ to 121 × 10^−5^ s^−1^ [52]. Similarly, the activation energy is estimated for the whole torrefaction process [53,54]. The average activation energy (*E_a_*) for the tested pruned biomass of *Paulownia clon* in Vitro was 36.5 kJ·mol^−1^. Díaz et al. [55] determined the values of activation energy for the individual constituents of *Paulownia* biomass. The *E_a_* values of cellulose, lignin, and hemicellulose were 10.4 kJ·mol^−1^, 17.2 kJ·mol^−1^, and 23.5 kJ·mol^−1^, respectively. Nevertheless, these values were determined for a wider (and higher) temperature range of 200–700 °C [55,56]. The obtained *E_a_* value for Oxytree is low in comparison with other types of woody biomass. For example, *E_a_* values for beech and spruce were 150 kJ·mol^−1^ and 155 kJ·mol^−1^ [48], 46–152 kJ·mol^−1^ for willow [57], 131 kJ·mol^−1^ for pine, and 128 kJ·mol^−1^ for fir [58]. Bach et al. [59] tested biomass fuels (Norwegian spruce and birch) in isothermal conditions at 230–280 °C (interval 10 °C) for 4 h, and used a two-step kinetic model. At first step, *E_a_* was 48.1 kJ·mol^−1^ and 55.1 kJ·mol^−1^, for spruce and birch, respectively, whereas, at the second step, *E_a_* was much higher, i.e., 99.2 kJ·mol^−1^ and 94.4 kJ·mol^−1^, respectively [59]. The *Paulownia* wood was co-pyrolysed with plastic waste by Chen et al. [60]. The use of the Kissinger–Akahira–Sunose (KAS) and Flynn–Wall–Ozawa (FWO) methods resulted in *E_a_* ranging from 167 to 189 kJ·mol^−1^. However, these *E_a_* values were determined by different methods and models. Thus, there is a need for a more unified approach to methods and reporting.

The same methodology of kinetics parameters determination as reported in this manuscript was used previously by Pulka et al. [61] and Syguła et al. [62] for sewage sludge and spent mushroom compost (MSC), respectively. For these materials, *E_a_* was 12 kJ·mol^−1^ and 22.2 kJ·mol^−1^, and *k* values ranged from 4.02 × 10^−5^ to 6.71 × 10^−5^ s^−1^ and 1.7 × 10^−5^ to 6.3 × 10^−5^ s^−1^ for sludge and MSC, respectively.

The kinetic model used in this research is a generic model. This model only allows estimating the yield at a specific temperature and time of the process. Additional studies on the kinetics of the process with a more complex model (e.g., incorporating improved mass and energy balance, gases and tars formed in the process) is warranted. Such information will allow scaling up and designing the technological line for the *Paulownia clon* in Vitro 112 torrefaction.

## 5. Conclusions

Research on the torrefaction kinetics of pruned biomass of Oxytree allowed us to conclude that:

- the cultivation method and soil type did not have an effect on the relative mass loss, *k* value, and energy activation (*p* < 0.05);
- the relative mass loss increased with the torrefaction temperature increase (as it is commonly reported for other types of biowaste torrefaction). The smallest relative mass loss was recorded at 200 °C, 10 min and the highest at 300 °C, 60 min, respectively 0.1% and 19.6% (*p* < 0.05);
- the constant reaction rate (*k* value) significantly increased with the torrefaction temperature increase. The smallest *k* value was 1.26 × 10^−5^ s^−1^, while the largest was 7.96 × 10^5^ s^−1^ at 200 °C and 300 °C, respectively;
- the average energy activation of the torrefaction in 200–300 °C was 36.5 kJ·mol^−1^.

The cultivation method (soil type, irrigation, geotextile use) makes no difference for the torrefaction process (lack of statistical differences for *E_a_* and *k* value at *p* < 0.05). Thus, Oxytree growers can focus on cultivation practices resulting in the most significant biomass yield. The results of this research, especially the kinetics parameters, make it possible to calculate the mass yield of a reliable biochar product for any process temperature and time, which is very useful from a practical point of view—the best biomass properties on sandy soil were with geotextile and without irrigation, on clay soil without geotextile and with irrigation.

Results of this work are complementary with previous articles [23,26] and, taken together, provide essential data about the torrefaction process of pruned Oxytree biomass. The next research questions are with respect to the gas products emitted from the torrefaction process and improved economics and environmental impact analyses.

## Figures and Tables

**Table 1:** Proximate analysis of pruned Oxytree biomass; mean values ± standard deviation.

Cultivation Type Symbol, -	Moisture Content, %	Organic Matter Content in d.m., %	Combustible Content in d.m., %	Ash in d.m., %	HHV, J·g d.m.^−1^	LHV, J·g d.m.^−1^
S(G−)(I−)	4.64 ± 0.09	89.22 ± 0.39	91.22 ± 0.38	8.78 ± 0.38	18,251 ± 90	16,666 ± 90
S(G+)(I−)	5.84 ± 0.04	90.36 ± 0.07	92.26 ± 0.09	7.74 ± 0.09	18,577 ± 195	17,135 ± 195
S(G−)(I+)	5.96 ± 0.02	90.10 ± 0.33	91.91 ± 0.29	8.09 ± 0.29	18,407 ± 199	16,762 ± 199
S(G+)(I+)	5.77 ± 0.05	90.06 ± 0.44	91.56 ± 0.44	8.44 ± 0.44	18,499 ± 69	16,858 ± 69
C(G+)(I−)	5.22 ± 0.01	91.00 ± 0.33	92.61 ± 0.29	7.39 ± 0.29	18,453 ± 135	16,920 ± 135
C(G−)(I−)	5.04 ± 0.01	89.75 ± 0.22	91.55 ± 0.21	8.45 ± 0.21	18,099 ± 441	16,463 ± 441
C(G+)(I+)	5.62 ± 0.04	89.86 ± 0.30	91.32 ± 0.31	8.68 ± 0.31	17,910 ± 407	16,430 ± 407
C(G−)(I+)	5.57 ± 0.01	91.46 ± 0.28	93.05 ± 0.18	6.95 ± 0.18	18,167 ± 377	16,450 ± 377
Mean	5.46 ± 0.43	90.23 ± 0.73	91.93 ± 0.67	8.07 ± 0.67	18,295 ± 317	16,711 ± 336

**Table 2:** Ultimate analysis of pruned Oxytree biomass, mean values ± standard deviation.

Cultivation Type Symbol, -	C, %	H, %	N, %	S, %	O, %
S(G−)(I−)	44.20	6.74	2.05	0.17	36.06
S(G+)(I−)	44.10	5.95	1.95	0.20	38.16
S(G−)(I+)	44.90	6.87	2.22	0.21	35.90
S(G+)(I+)	45.10	6.87	2.83	0.20	35.06
C(G+)(I−)	44.10	6.44	2.72	0.21	37.53
C(G−)(I−)	40.20	6.93	2.46	0.22	39.95
C(G+)(I+)	41.80	6.15	2.06	0.19	39.66
C(G−)(I+)	45.70	7.24	2.09	0.19	36.23
Mean	43.76 ± 1.84	6.65 ± 0.43	2.30 ± 0.33	0.20 ± 0.02	37.32 ± 1.81

**Table 3:** Relative mass loss (Δ*m*) of pruned Oxytree biomass during torrefaction.

Temperature, °C	Time, min	Relative Mass Loss, %							
		S(G−)(I−)	S(G+)(I−)	S(G−)(I+)	S(G+)(I+)	C(G+)(I−)	C(G−)(I−)	C(G+)(I+)	C(G−)(I+)
200	10	0.1 ± 0.2	0.2 ± 0.2	0.2 ± 0.2	0.4 ± 0.2	0.1 ± 0.2	0.1 ± 0.2	0.2 ± 0.2	0.1 ± 0.2
	20	0.2 ± 0.4	0.3 ± 0.0	0.4 ± 0.2	0.9 ± 0.2	0.4 ± 0.2	0.4 ± 0.2	0.6 ± 0.2	0.4 ± 0.2
	30	0.4 ± 0.5	0.7 ± 0.0	0.7 ± 0.3	1.3 ± 0.3	0.6 ± 0.2	0.8 ± 0.2	0.9 ± 0.2	0.8 ± 0.2
	40	0.8 ± 0.5	0.9 ± 0.2	1.0 ± 0.3	1.8 ± 0.2	0.8 ± 0.2	1.0 ± 0.0	1.0 ± 0.0	1.0 ± 0.0
	50	1.0 ± 0.7	1.0 ± 0.0	1.2 ± 0.5	2.1 ± 0.5	1.0 ± 0.0	1.2 ± 0.2	1.3 ± 0.2	1.2 ± 0.2
	60	1.2 ± 0.5	1.3 ± 0.0	1.4 ± 0.7	2.4 ± 0.5	1.2 ± 0.2	1.3 ± 0.0	1.6 ± 0.0	1.4 ± 0.2
220	10	0.0 ± 0.0	0.3 ± 0.3	0.2 ± 0.2	0.3 ± 0.2	0.4 ± 0.2	0.2 ± 0.2	0.4 ± 0.2	0.3 ± 0.0
	20	0.1 ± 0.2	0.8 ± 0.7	0.7 ± 0.3	1.2 ± 0.2	1.2 ± 0.2	0.8 ± 0.7	1.1 ± 0.2	1.2 ± 0.2
	30	0.6 ± 0.7	1.6 ± 0.8	1.3 ± 0.3	2.1 ± 0.2	1.9 ± 0.2	1.8 ± 0.7	2.0 ± 0.3	2.0 ± 0.3
	40	1.3 ± 0.9	2.2 ± 0.8	1.9 ± 0.5	2.8 ± 0.2	2.3 ± 0.0	2.6 ± 0.8	2.8 ± 0.2	3.0 ± 0.3
	50	2.0 ± 0.9	2.9 ± 0.8	2.3 ± 0.3	3.4 ± 0.2	2.9 ± 0.2	3.2 ± 0.8	3.2 ± 0.2	3.6 ± 0.4
	60	2.7 ± 1.2	3.3 ± 0.7	2.7 ± 0.3	4.0 ± 0.0	3.2 ± 0.2	3.7 ± 0.6	3.7 ± 0.3	4.1 ± 0.5
240	10	0.3 ± 0.0	0.8 ± 0.2	0.4 ± 0.0	0.8 ± 0.2	0.6 ± 0.4	0.9 ± 0.2	0.7 ± 0.0	0.4 ± 0.4
	20	1.4 ± 0.2	2.2 ± 0.2	1.8 ± 0.2	2.7 ± 0.2	1.9 ± 1.0	2.6 ± 0.5	2.2 ± 0.2	2.0 ± 0.9
	30	2.9 ± 0.2	4.0 ± 0.3	3.3 ± 0.2	4.7 ± 0.3	3.7 ± 1.5	4.3 ± 0.7	4.2 ± 0.2	3.9 ± 0.8
	40	4.2 ± 0.2	5.3 ± 0.3	4.8 ± 0.2	6.0 ± 0.3	4.9 ± 1.3	5.7 ± 0.7	5.3 ± 0.3	5.3 ± 0.3
	50	5.2 ± 0.2	6.2 ± 0.2	5.9 ± 0.2	7.0 ± 0.2	5.8 ± 1.1	6.3 ± 0.7	6.0 ± 0.3	6.4 ± 0.2
	60	5.9 ± 0.2	6.8 ± 0.2	6.6 ± 0.2	7.4 ± 0.2	6.3 ± 0.8	6.8 ± 0.8	6.4 ± 0.4	7.0 ± 0.3
260	10	0.3 ± 0.3	1.2 ± 0.2	1.2 ± 0.2	1.0 ± 0.7	1.1 ± 0.4	1.8 ± 0.2	1.1 ± 0.4	1.2 ± 0.2
	20	1.7 ± 0.9	4.2 ± 0.2	4.3 ± 0.3	3.9 ± 1.4	4.1 ± 0.4	6.0 ± 0.3	4.4 ± 0.4	5.1 ± 0.2
	30	4.1 ± 1.5	7.2 ± 0.2	7.4 ± 0.4	6.9 ± 1.3	6.7 ± 0.3	8.8 ± 0.2	6.8 ± 0.4	7.8 ± 0.2
	40	5.7 ± 1.5	8.7 ± 0.0	9.0 ± 0.3	8.3 ± 1.2	8.0 ± 0.0	10.3 ± 0.3	8.0 ± 0.3	9.1 ± 0.2
	50	6.7 ± 1.5	9.6 ± 0.2	9.9 ± 0.5	9.4 ± 1.0	8.7 ± 0.0	11.3 ± 0.3	8.7 ± 0.3	9.9 ± 0.4
	60	7.4 ± 1.6	10.0 ± 0.0	10.3 ± 0.3	10.1 ± 0.7	9.2 ± 0.2	12.2 ± 0.4	9.1 ± 0.2	10.4 ± 0.2
280	10	0.8 ± 0.2	1.3 ± 0.9	2.1 ± 0.4	2.2 ± 0.2	2.1 ± 0.2	2.4 ± 0.2	1.9 ± 0.8	2.1 ± 0.4
	20	4.7 ± 0.0	6.7 ± 2.0	8.2 ± 0.5	8.2 ± 0.5	7.7 ± 0.3	8.7 ± 0.0	7.6 ± 1.3	8.3 ± 0.3
	30	8.4 ± 0.4	10.0 ± 1.2	11.3 ± 0.3	11.3 ± 0.3	10.4 ± 0.2	11.3 ± 0.0	10.1 ± 1.3	11.2 ± 0.4
	40	10.0 ± 0.3	11.7 ± 0.9	12.9 ± 0.2	12.8 ± 0.5	12.0 ± 0.3	12.6 ± 0.2	11.7 ± 1.2	12.8 ± 0.2
	50	11.1 ± 0.4	12.7 ± 0.7	14.0 ± 0.3	13.8 ± 0.5	13.0 ± 0.3	13.6 ± 0.2	12.6 ± 1.1	13.7 ± 0.3
	60	11.6 ± 0.5	13.3 ± 0.7	14.7 ± 0.3	14.3 ± 0.3	13.7 ± 0.3	14.2 ± 0.2	13.3 ± 0.9	14.3 ± 0.3
300	10	1.3 ± 0.9	2.9 ± 0.2	3.4 ± 0.2	3.6 ± 0.7	3.1 ± 1.3	3.4 ± 0.7	3.3 ± 0.0	2.9 ± 1.6
	20	8.3 ± 1.2	11.3 ± 0.3	12.0 ± 0.3	12.1 ± 0.5	10.7 ± 1.5	11.4 ± 0.7	11.1 ± 0.2	11.2 ± 1.9
	30	12.2 ± 1.1	14.7 ± 0.3	15.7 ± 0.3	15.7 ± 0.3	14.0 ± 1.2	14.9 ± 0.5	14.4 ± 0.2	14.7 ± 1.7
	40	14.3 ± 1.2	16.4 ± 0.4	17.7 ± 0.3	17.4 ± 0.4	15.8 ± 1.8	16.6 ± 0.5	16.1 ± 0.2	16.6 ± 1.6
	50	15.7 ± 1.2	17.7 ± 0.3	19.0 ± 0.3	18.8 ± 0.4	16.9 ± 0.8	17.7 ± 0.3	17.3 ± 0.3	17.9 ± 1.3
	60	16.6 ± 1.2	18.3 ± 0.3	19.8 ± 0.5	19.6 ± 0.2	17.7 ± 0.7	18.4 ± 0.4	18.0 ± 0.3	18.8 ± 1.3

**Table 4:** Kinetic parameters of torrefaction of pruned biomass of Oxytree.

Cultivation Type Symbol	Process Temperature		Constant Rate of the Reaction	Arrhenius Plot Parameters		Activation Energy	Determination Coefficient
-	T, °C	T, K	*k*, s^−1^	1/T, K^−1^	ln(*k*), s^−1^	*Ea*, J·mol^−1^	R^2^, -
S(G−)(I−)	200	473	1.30 × 10^−5^	2.11 × 10^−3^	−11.3	35,028	0.98
	220	493	1.95 × 10^−5^	2.03 × 10^−3^	−10.9		
	240	513	2.99 × 10^−5^	1.95 × 10^−3^	−10.4		
	260	533	3.24 × 10^−5^	1.88 × 10^−3^	−10.3		
	280	553	4.69 × 10^−5^	1.81 × 10^−3^	−9.97		
	300	573	6.64 × 10^−5^	1.75 × 10^−3^	−9.62		
S(G+)(I−)	200	473	1.59 × 10^−5^	2.11 × 10^−3^	−11.1	33,369	0.99
	220	493	2.21 × 10^−5^	2.03 × 10^−3^	−10.7		
	240	513	3.42 × 10^−5^	1.95 × 10^−3^	−10.3		
	260	533	4.29 × 10^−5^	1.88 × 10^−3^	−10.1		
	280	553	5.16 × 10^−5^	1.81 × 10^−3^	−9.87		
	300	573	7.21 × 10^−5^	1.75 × 10^−3^	−9.54		
S(G−)(I+)	200	473	1.44 × 10^−5^	2.11 × 10^−3^	−11.2	39,407	0.99
	220	493	1.97 × 10^−5^	2.03 × 10^−3^	−10.8		
	240	513	3.09 × 10^−5^	1.95 × 10^−3^	−10.4		
	260	533	4.56 × 10^−5^	1.88 × 10^−3^	−10.0		
	280	553	6.19 × 10^−5^	1.81 × 10^−3^	−9.69		
	300	573	7.70 × 10^−5^	1.75 × 10^−3^	−9.47		
S(G+)(I+)	200	473	1.82 × 10^−5^	2.11 × 10^−3^	−10.9	34,282	0.99
	220	493	2.13 × 10^−5^	2.03 × 10^−3^	−10.8		
	240	513	3.26 × 10^−5^	1.95 × 10^−3^	−10.3		
	260	533	4.28 × 10^−5^	1.88 × 10^−3^	−10.1		
	280	553	5.85 × 10^−5^	1.81 × 10^−3^	−9.75		
	300	573	7.96 × 10^−5^	1.75 × 10^−3^	−9.44		
C(G+)(I−)	200	473	1.26 × 10^−5^	2.11 × 10^−3^	−11.3	38,436	0.98
	220	493	1.81 × 10^−5^	2.03 × 10^−3^	−10.9		
	240	513	3.22 × 10^−5^	1.95 × 10^−3^	−10.3		
	260	533	4.15 × 10^−5^	1.88 × 10^−3^	−10.1		
	280	553	5.15 × 10^−5^	1.81 × 10^−3^	−9.87		
	300	573	6.86 × 10^−5^	1.75 × 10^−3^	−9.59		
C(G−)(I−)	200	473	1.39 × 10^−5^	2.11 × 10^−3^	−11.2	36,210	0.98
	220	493	2.31 × 10^−5^	2.03 × 10^−3^	−10.7		
	240	513	3.44 × 10^−5^	1.95 × 10^−3^	−10.3		
	260	533	4.63 × 10^−5^	1.88 × 10^−3^	−9.98		
	280	553	5.37 × 10^−5^	1.81 × 10^−3^	−9.83		
	300	573	7.36 × 10^−5^	1.75 × 10^−3^	−9.52		
C(G+)(I+)	200	473	1.34 × 10^−5^	2.11 × 10^−3^	−11.2	36,442	0.99
	220	493	1.78 × 10^−5^	2.03 × 10^−3^	−10.9		
	240	513	2.72 × 10^−5^	1.95 × 10^−3^	−10.5		
	260	533	3.46 × 10^−5^	1.88 × 10^−3^	−10.3		
	280	553	4.95 × 10^−5^	1.81 × 10^−3^	−9.91		
	300	573	6.66 × 10^−5^	1.75 × 10^−3^	−9.62		
C(G−)(I+)	200	473	1.29 × 10^−5^	2.11 × 10^−3^	−11.3	38,907	0.99
	220	493	2.17 × 10^−5^	2.03 × 10^−3^	−10.7		
	240	513	3.39 × 10^−5^	1.95 × 10^−3^	−10.3		
	260	533	4.57 × 10^−5^	1.88 × 10^−3^	−9.99		
	280	553	5.61 × 10^−5^	1.81 × 10^−3^	−9.79		
	300	573	7.57 × 10^−5^	1.75 × 10^−3^	−9.49		

**Figure A1:**
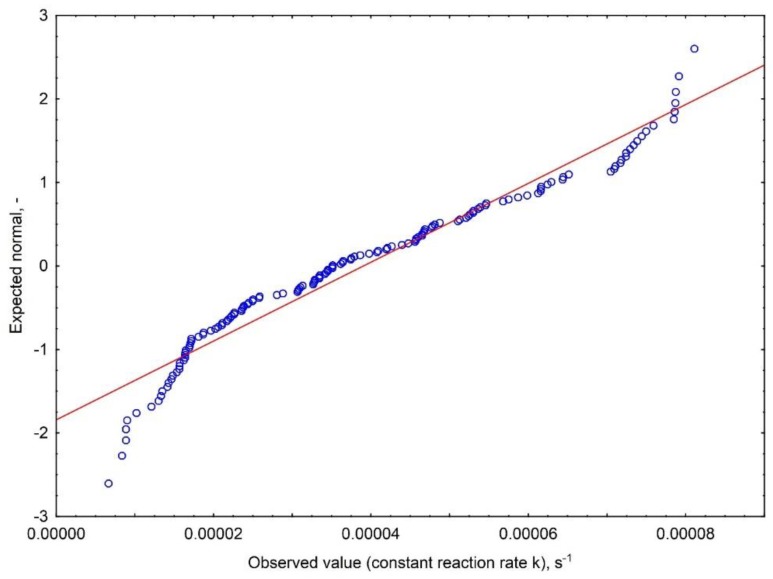
Normality plot of constant reaction rate *k.*

**Figure A2:**
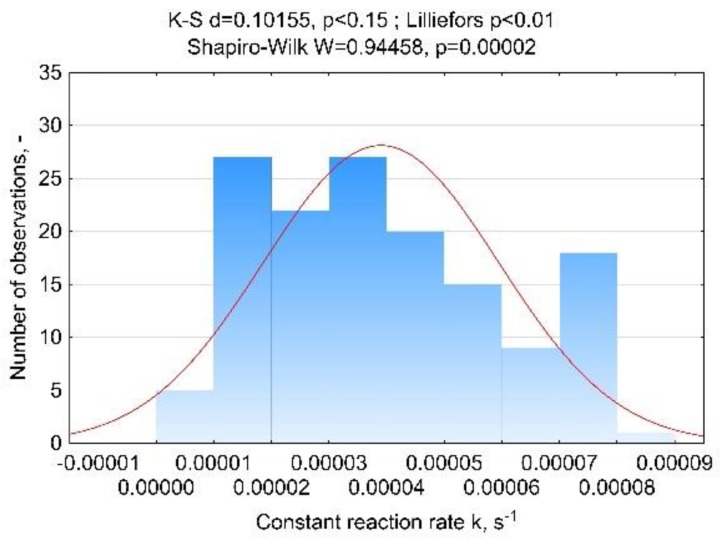
Histogram and the values of K.S., K.S. with the Lilliefors amendment, and S.W. tests for *k.*

**Figure A5:**
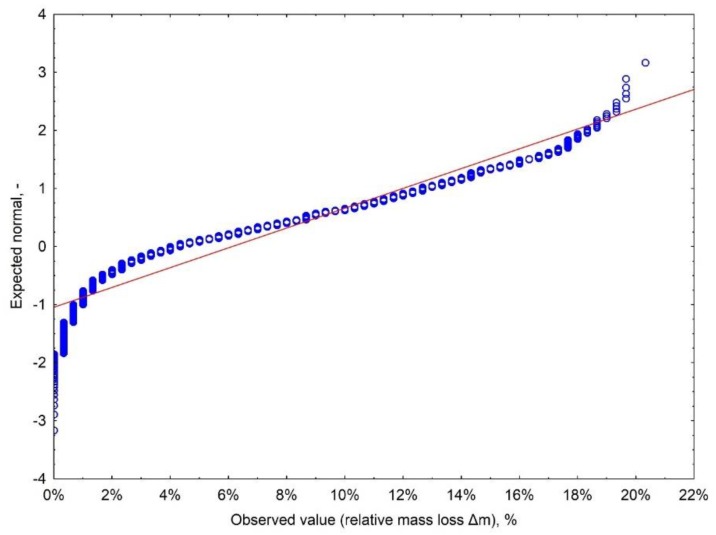
Quantile-quantile plot for Δ*m* during torrefaction of Oxytree.

**Figure A6:**
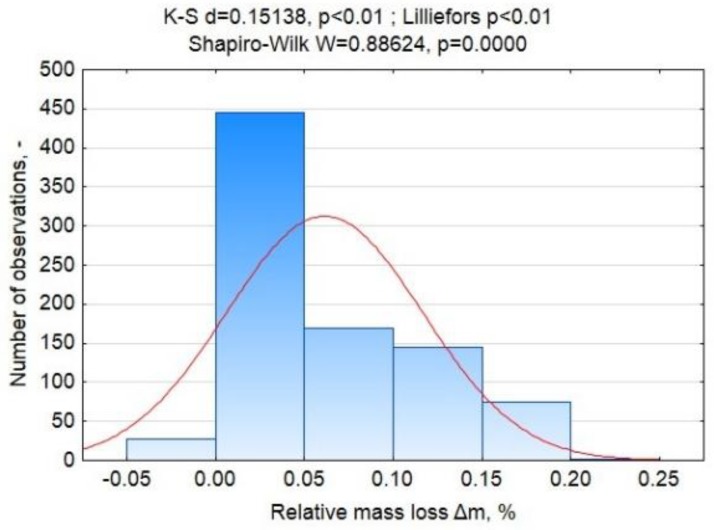
Histogram and the values of K.S., K.S. with the Lilliefors amendment, and S.W. tests for Δ*m* during torrefaction of Oxytree.

**Figure A15:**
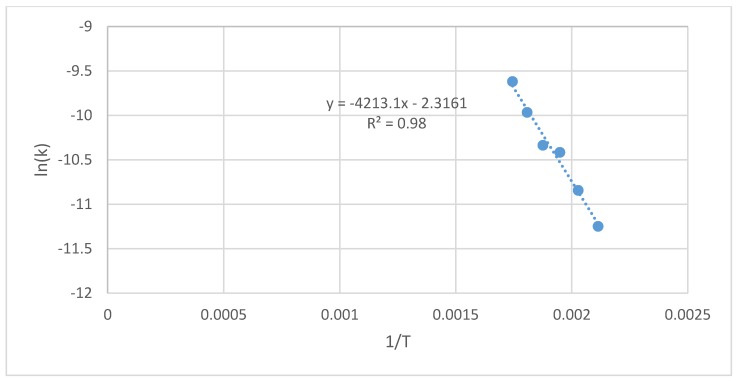
Arrhenius plot for S(G−)(I−).

**Figure A16:**
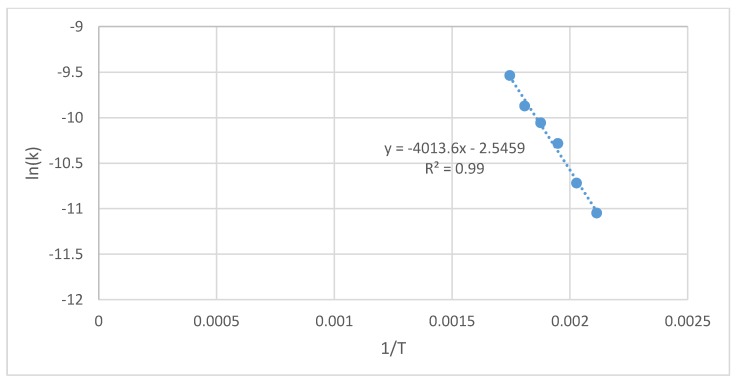
Arrhenius plot for S(G + )(I−).

**Figure A17:**
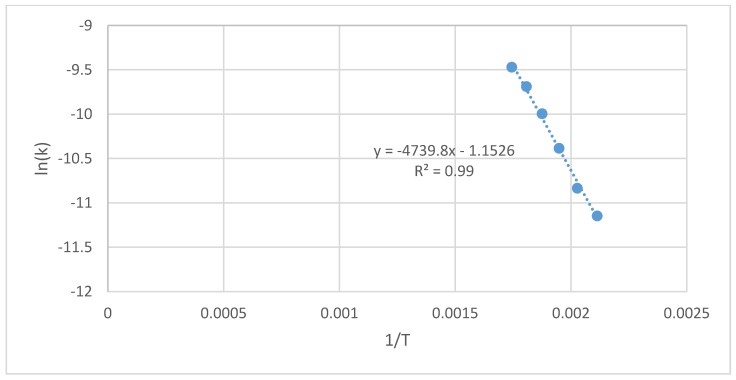
Arrhenius plot for S(G−)(I + ).

**Figure A18:**
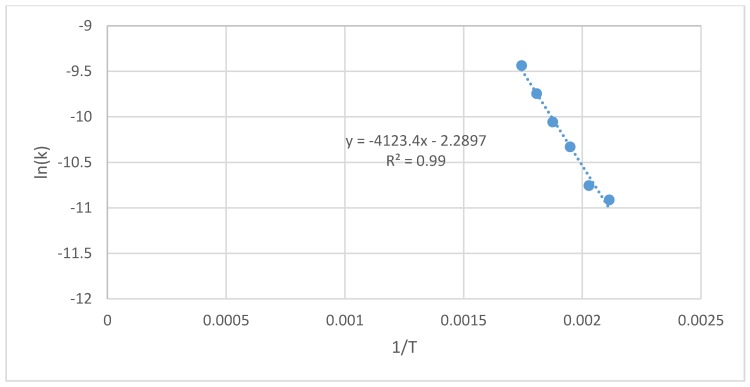
Arrhenius plot for S(G + )(I + ).

**Figure A19:**
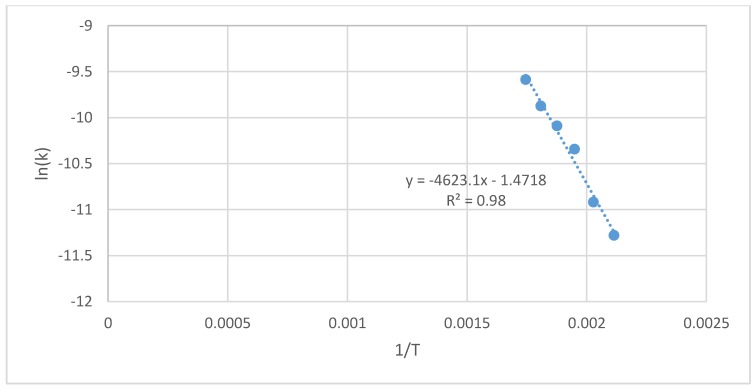
Arrhenius plot for C(G + )(I−).

**Figure A20:**
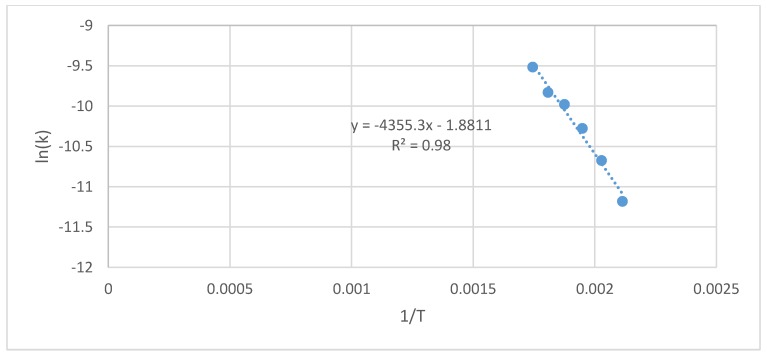
Arrhenius plot for C(G−)(I−).

**Figure A21:**
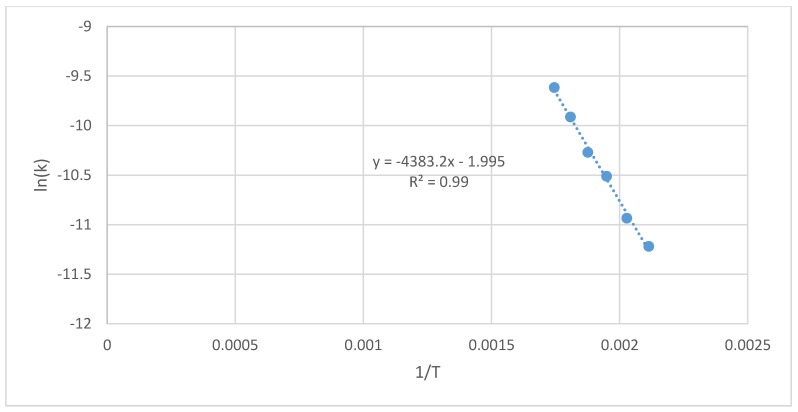
Arrhenius plot for C(G + )(I−).

**Figure A22:**
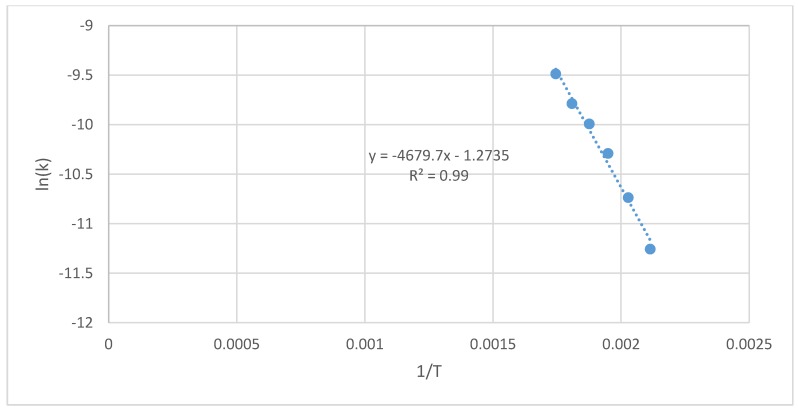
Arrhenius plot for C(G−)(I + ).

**Figure A23:**
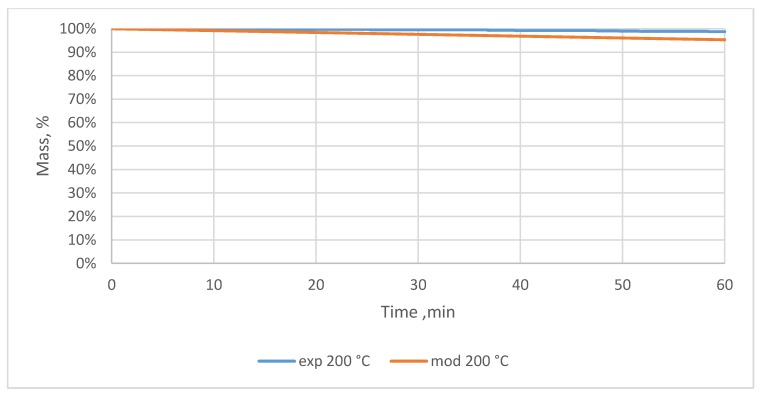
Mass losses during torrefaction at 200 °C; material S(G−)(I−).

**Figure A24:**
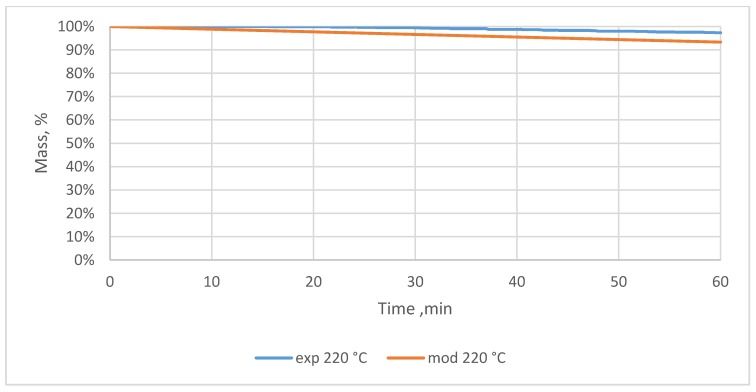
Mass losses during torrefaction at 220 °C; material S(G−)(I−).

**Figure A25:**
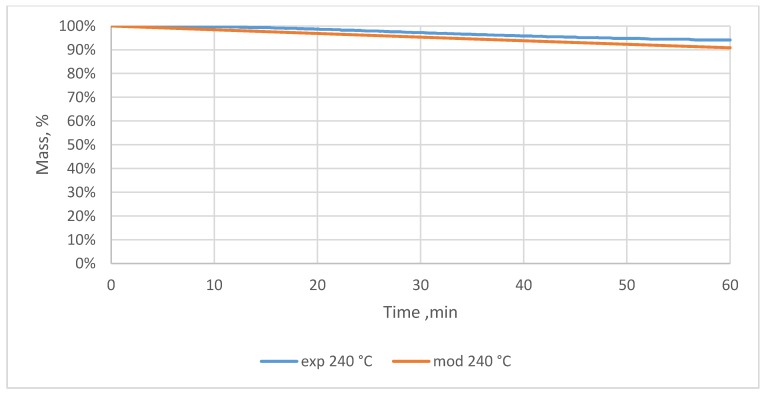
Mass losses during torrefaction at 240 °C; material S(G−)(I−).

**Figure A26:**
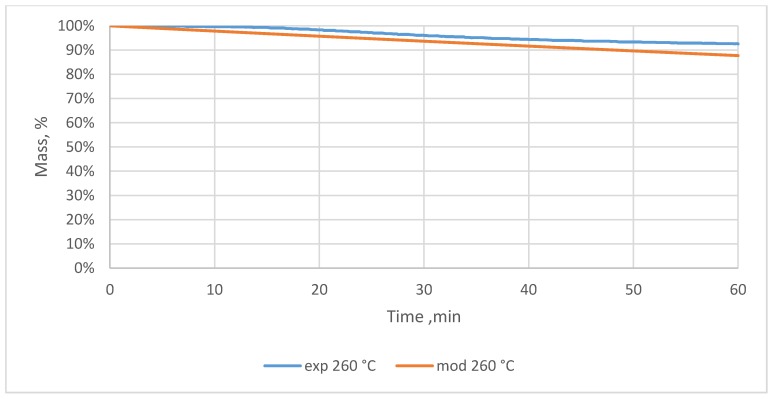
Mass losses during torrefaction at 260 °C; material S(G−)(I−).

**Figure A27:**
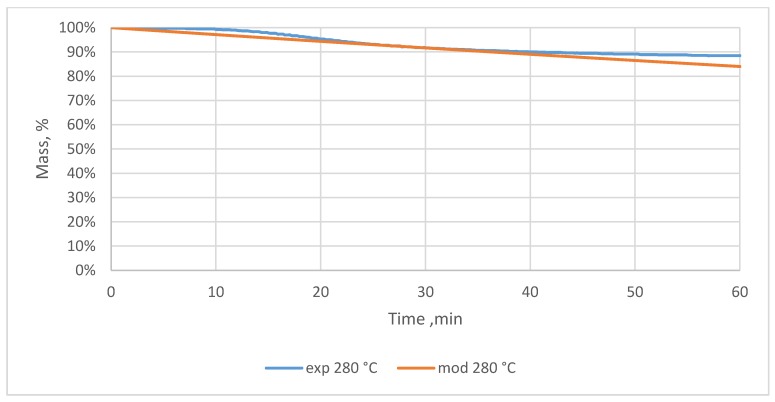
Mass losses during torrefaction at 280 °C; material S(G−)(I−).

**Figure A28:**
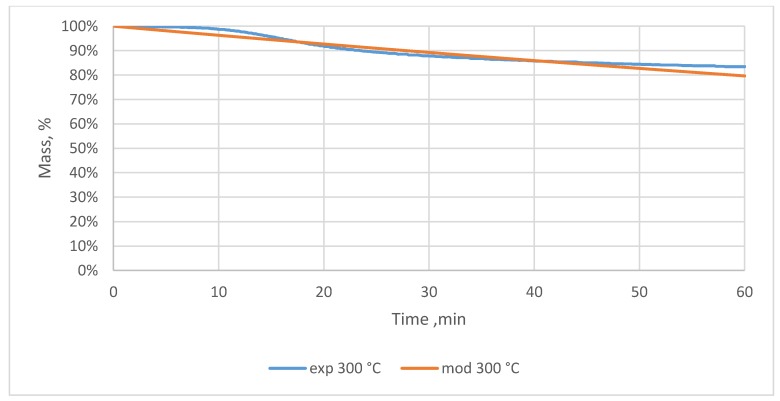
Mass losses during torrefaction at 300 °C; material S(G−)(I−).

## Appendix A

### Appendix A.1. Statistical Evaluation of the k Value

#### Appendix A.1.1. Results of Distribution Normality Evaluation

The study of the normality of the distribution of *k* values was performed graphically using a normality plot (Figure A1) and Shapiro–Wilk (S.W.) and Kolmogorov–Smirnov (K.S.) statistical tests, along with the Lilliefors correction for α = 0.05 (Figure A2). The following hypotheses were assumed for both tests:

**Hypothesis** **0 (H0).**
*The distribution of k values is a normal distribution.*

**Hypothesis** **1 (H1).**
*The k value does not have a normal distribution.*

#### Appendix A.1.2. Analysis of Variance

Since the distribution of *k* values was not a normal distribution, the analysis of variance was carried out with the non-parametric Kruskal–Wallis test (K.W.). Two non-parametric tests (K.W.) were carried out at the level of α = 0.05:

- *k* relative to the variable grouping the cultivation type;
- *k* relative to the variable that groups the process temperature.

The tests assumed hypotheses 0 showing no influence of the grouping variable on the value of *k* and hypothesis 1 showing the influence of the grouping variable on the *k* value. Multiple comparisons (post-hoc test) were performed to determine which groups had statistically significant differences. The lack of significant differences was marked with the same letters on the box plots.

#### Appendix A.1.3. Results and Interpretation

The *k* values did not have a normal distribution because the points did not lie in one line, as shown on the normality plot (Figure A1). Results of the S.W. test show that that *p* = 0.00002 and < α = 0.05 so hypothesis 0 on the normality of the distribution should be rejected. The K.S. test (Figure A2) shows that the distribution was normal, because *p* = 0.15 > α = 0.05. On the other hand, the Lilliefors amendment (Figure A2), which takes into account the lack of knowledge of the mean value and standard deviation of the population, shows that hypothesis 0 should be rejected because *p* = 0.01 and <α = 0.05.

The test value (K.W.) for *k* relative to the variable grouping for the cultivation type was *H* = 2.689 and *p* = 0.912. Since *p* = 0.912 > α = 0.05 should be considered a valid hypothesis 0, this means that the cultivation type *Paulownia clon* in Vitro 112 did not have a statistically significant impact on the *k* value. The lack of statistical differences obtained from the K.W. test is illustrated by the same letters in Figure A3. Figure A3 presents the average *k* values for individual cultivation types with the standard deviation and the standard error. The highest average value of *k* was determined for the S(G+)(I−) and S(G−)(I+) materials, and the lowest *k* for S(G−)(I−) (not significantly different).

The K.W. test value for *k* in relation to the variable grouping for the temperature was *H* = 133.386 and *p* = 0.000. Since *p* = 0.000 < α = 0.05, hypothesis 1 stands. This means that the process temperature had a statistically significant impact on the *k* value. The statistical differences obtained from the K.W. test are illustrated by different letters in Figure A4. The graph presents the mean values of *k* with respect to temperature (Figure A4). The lowest average *k* value was recorded at 200 °C, i.e., <2 × 10^−5^ s^−1^, and the highest at 300 °C, i.e., >7 × 10^−5^ s^−1^. The post-hoc test showed that statistically significant differences occurred approximately every 40 °C. The lack of significant differences (*p* < 0.05) is marked with the same letters.

### Appendix A.2. Relative Mass Loss Δm, %

#### Appendix A.2.1. Results of the Distribution Normality Evaluation

The study of Δ*m* distribution was performed graphically using a normality plot (Figure A5) and statistical tests of S.W. and K.S. along with the Lilliefors correction for α = 0.05 (Figure A6). The following hypotheses were assumed for both tests:

**Hypothesis** **0 (H0).**
*The distribution of Δm values was a normal distribution.*

**Hypothesis** **1 (H1).**
*The Δm value did not have a normal distribution.*

Since the distribution of Δ*m* did not have a normal distribution, the analysis of variance was carried out with the non-parametric K.W. test.

#### Appendix A.2.2. Analysis of Variance

Three non-parametric tests (K.W.) were carried out at the level of α = 0.05:

- Δ*m* relative to the variable grouping the cultivation type;
- Δ*m* relative to the variable grouping process temperatures;
- Δ*m* relative to the variable that groups the process time.

#### Appendix A.2.3. Results and Interpretation

The Δ*m* values did not have a normal distribution because the points did not lie in one line (Figure A5). The S.W. test showed that *p* = 0.0000 < α = 0.05, so hypothesis 0 on the normality of the distribution was rejected. The K.S. test showed that the distribution was not normal because *p* = 0.01 > α = 0.05. Lilliefors’ test also confirmed that hypothesis 0 should be rejected because *p* = 0.01 < α = 0.05 (Figure A6).

The K.W. test value for Δ*m* relative to the grouping variable of the cultivation type was *H* = 12.299 and *p* = 0.0912. Since *p* = 0.0912 > α = 0.05, the null hypothesis should be considered as the correct one. This means that the cultivation type had no statistically significant effect on Δ*m* during torrefaction. Figure A7 presents the average Δ*m* values for particular cultivation types along with the standard deviation. The highest mean Δ*m* was measured for S(G-)(I+), S(G+)(I+) and C(G-)(I-), and the smallest for S(G-)(I-). As the K.W. test showed, these differences were not statistically significant (*p* < 0.05).

The K.W. test value for Δ*m* relative to the variable grouping the process temperature was *H* = 543.953 and *p* = 0.000. Because *p* = 0.000 < α = 0.05, hypothesis 1 should be considered the correct one. It means that the process temperature affected the Δ*m*. The post-hoc test showed that statistically significant differences existed between all torrefaction temperature setpoints used in the study (*p* < 0.05). The average Δ*m* values for individual temperatures are shown in Figure A8. The lowest Δ*m* occurred at 200 °C (0%–2%), and the highest at 300 °C (8%–19%), depending on the torrefaction time.

The K.W. test value for Δ*m* relative to the variable grouping the process time was *H* = 258.684 and *p* = 0.000. Since *p* = 0.000 < α = 0.05, hypothesis 1 should be considered as the correct one. This means that the torrefaction time affected Δ*m*. The post-hoc test showed that statistically significant differences did not exist between any of the time groups. Groups with no statistically significant differences (*p* < 0.05) were marked with the same letter in Figure A9. Time had a significant effect on Δ*m* for up to 30 min of torrefaction. The average Δ*m* values did not differ significantly from 30 to 50 min. The value of Δm at 30 min was significantly different from the value of Δ*m* at 60 min, while the values in 40 and 50 min did not differ significantly from the values obtained at 60 min.

### Appendix A.3. Oxytree Biomass Characterization

#### Appendix A.3.1. Results of the Distribution Normality Evaluation

Due to the fact that only three replicates were completed to determine each parameter, there was no reason to check the distribution of normality (too small of a sample group). Thus, the lack of normality distributions was assumed, similarly as in previous parameters (presented above).

#### Appendix A.3.2. Analysis of Variance

Three non-parametric K.W. tests were carried out at the level of α = 0.05;

- Organic matter content relative to the variable grouping of the cultivation type;
- Ash relative to the variable grouping of the cultivation type;
- Combustible content relative to the variable grouping of the cultivation type;
- High heating value relative to the variable grouping of the cultivation type; and
- Low heating value relative to the variable grouping of the cultivation type.

In the tests, it was assumed that:

- Hypotheses 0 showed that the grouping variable does not affect the Δ*m*; and
- Hypothesis 1 tests the influence of the grouping variable on the Δ*m* value.

In order to determine which variable groupings had statistically significant differences, multiple comparisons (post-hoc tests) were performed. The lack of significant differences was marked with the same letters on the box plot.

#### Appendix A.3.3. Results and Interpretation

The post-hoc K.W. tests were carried out in relation to basic biomass parameters obtained on different cultivation methods. The tests showed differences between the effects of the cultivation method on organic matter content (Figure A10), combustible content (Figure A11), and ash content (Figure A12). No statistical differences were observed with respect to *LHV* and *HHV* (Figure A13 and Figure A14).

## Appendix B

The Arrhenius plots (Figure A15, Figure A16, Figure A17, Figure A18, Figure A19, Figure A20, Figure A21 and Figure A22) for each tested material are presented. The figures illustrate the linear model with a determination coefficient (R^2^). The activation energy was determined based on the slope coefficient.

Figure A23, Figure A24, Figure A25, Figure A26, Figure A27 and Figure A28 present differences between experimental (exp) and calculated (mod) mass loss during torrefaction at setpoint temperatures.
